# Dual-band composite right/left-handed metamaterial lines with dynamically controllable nonreciprocal phase shift proportional to operating frequency

**DOI:** 10.1515/nanoph-2021-0783

**Published:** 2022-04-07

**Authors:** Takumi Kaneda, Tetsuya Ueda

**Affiliations:** Electrical Engineering and Electronics Department, Kyoto Institute of Technology, 1 Hashigami-cho, Matsugasaki, Sakyo-ku, Kyoto 606-8585, Japan

**Keywords:** dual band, metamaterials, nonreciprocal circuits, periodic structures, phase shifters, unidirectional traveling wave resonators

## Abstract

Dual-band composite right/left-handed transmission lines with the nonreciprocal phase shift approximately proportional to the operating frequency are proposed and demonstrated by using normally magnetized ferrite microstrip lines. The nonreciprocal phase shift can be dynamically controlled by changing the externally applied dc magnetic field. Dynamic change in the nonreciprocal phase gradient along the line enables us to realize dual-band and unidirectional beam-scanning leaky-wave antennas without suffering from frequency-dependent change in the beam direction. A prototype nonreciprocal metamaterial line using polycrystalline yttrium iron garnet was fabricated and measured for verification of our basic concept.

## 1 Introduction

Metamaterials have been studied and developed in various kinds of applications where the technology enables us to design macroscopic constitution relations for artificial structures/circuits with unit cells of the subwavelength scale. Composite right/left-handed (CRLH) transmission lines [1, 2] are one of metamaterials based on circuit theory, and the basic concept can apply not only to microwave/millimeter wave and terahertz wave engineering, but also to infrared and even optical range to interpret various electromagnetic phenomena and fundamental mechanism of wave propagation. The CRLH transmission line can have two independent resonant systems in series and shunt branches of the unit cell with resonant frequencies corresponding to zero effective permittivity or zero permeability, at which we can design transmission-line resonators with operating frequencies independent of the resonators’ size, referred to as zeroth-order resonators having uniform field profiles along the resonators [3]. When the two resonant frequencies at zero-permittivity and permeability are the same, a bandgap appearing in the frequency region between these resonant frequencies can be eliminated to accidentally form a Dirac cone with an intersection of dispersion curves, that is, a Dirac point appearing on the frequency axis [4], [5], [6], [7]. This condition is equivalent to a matching condition of two impedances between the transmission line and the inserted lumped elements [4, 5]. Dual-band, or extended CRLH metamaterial lines are also proposed [8], [9], [10], [11], [12], [13], [14], [15], and the design procedures to form two Dirac points were also given for reciprocal cases [9].

Nonreciprocity to be discussed here means that transmission coefficients along 1-D waveguides or transmission lines have the differences between two anti-parallel directions of wave propagation. In general, the nonreciprocity originates from a combination of both broken time-reversal and space-inversion symmetries. To realize time-reversal asymmetry, gyro-tropic materials such as ferromagnetic and ferrite materials in the microwave/millimeter-wave regions and magneto-optic media in optics have been utilized, and more recently the operating regions have got into terahertz region [16, 17]. In some cases, the nonreciprocal phenomena are found in differences of magnitudes of the coefficients that are promising for applications to isolators and circulators. In some other cases, the nonreciprocity significantly appears in the argument or phase shift of the complex transmission coefficients, namely in the phase constants (equivalent to “wavevectors”) or in the effective refractive indices. Then the magnitudes of the transmission coefficients may possess almost identical characteristics.

When nonreciprocal phase-shift in transmission characteristics is additionally provided to CRLH metamaterial lines, such as by using geometrically asymmetric and normally magnetized ferrite microstrip lines which supports edge guided mode propagation due to field displacement effect [18, 19], position of the Dirac point can be moved along the horizontal phase constant axis, to the left or right away from the vertical frequency axis [20, 21]. As a result, we can design nonreciprocal CRLH lines with positive refractive index in one propagation direction and negative refractive index in the opposite direction at the same frequency [20]. Then the round-trip propagation along such nonreciprocal CRLH lines operating at the Dirac point automatically satisfy the phase-matching condition, i.e., resonant condition independently of the resonators’ size, and the resonance shows fascinating field profiles with the uniform magnitude and nonzero phase gradient along the resonator, which lead to manifestation of size-independent unidirectional traveling wave resonances [22]. If the resonant mechanism operates in the “fast wave region,” i.e., inside the light cone where the phase velocity exceeds the speed of light in vacuum, the resonator gives rise to highly efficient unidirectional beam-forming leaky wave radiation whose beam direction is determined by the nonzero phase constants at the Dirac point and dynamically controlled by changing the externally applied dc magnetic field to the ferrites [22]. The nonreciprocal metamaterial resonators were implemented to compact and highly efficient beam-scanning leaky-wave antennas with frequency-independent beam direction [22, 23], or additional functionality of polarization-plane rotation by changing the end conditions [24].

Conventional leaky wave antennas using one-way traveling wave propagation are frequency-selective beam scanning antennas with very simple configuration, and even electronically controlled beam-scanning antennas may suffer from “beam squint” problem in which beam direction varies with the operational frequency. There have been many publications on beam squint reduction techniques in the leaky wave antennas, such as by using frequency-dispersive metasurfaces [25] or metamaterial lenses [26] as the superstates that were placed above the original leaky wave antennas to compensate the dispersive characteristics. Another approach practically realized ideal, dispersion-less, or linear dispersion curves in the fast wave region by using active elements with gains, such as non-Foster circuits [27, 28], in which the group velocity exceeds the speed of light in vacuum in the fast wave region. However, this kind of approach requires high electromagnetic power consumption to achieve the performance. If passive-circuit-based nonreciprocal CRLH metamaterial lines are designed so that the nonreciprocal phase-shift is proportional to the operational frequency, the beam direction of the leaky waves from the unidirectional traveling-wave resonators can be kept constant in the vicinity of the resonant frequency. That is, the beam squint can be eliminated based on the passive circuits. However, the nonreciprocal-metamaterial-based beam squint reduction technique was applied only to the single-band operation. More recently, dual-band nonreciprocal CRLH metamaterial lines were designed with two Dirac points [29]. However, the nonreciprocal phase-shift at dual bands show a very complicated frequency dependence and do not have the linear relation. As a result, leaky waves radiated at lower and higher operating frequencies go to different directions. If the two Dirac points have the nonreciprocal phase-shift proportional to the operating frequency, the beam angles at the dual bands can achieve a unidirectional direction. In addition, if both tangents of the nonreciprocities at the dual bands are proportional to the frequency, beam squint can also be eliminated in the vicinity of the operation frequencies.

In this paper, we propose and demonstrate dual-band nonreciprocal CRLH metamaterial lines having two Dirac points with nonreciprocal phase shift approximately proportional to operating frequencies by using normally magnetized ferrite microstrip lines. The nonreciprocal phase shift can be dynamically controlled by changing the externally applied dc magnetic field to the ferrite. Dynamic change in the nonreciprocal phase gradient of the fields along the line enables us to simultaneously realize dual-band, unidirectional, and squint-free beam-scanning leaky-wave antennas. A prototype nonreciprocal CRLH metamaterial line using polycrystalline yttrium iron garnet was fabricated and the transmission characteristics were measured for verification of our basic concept.

## 2 Basic concept

In this section, we will explain why we need to design and control a frequency dependence of the nonreciprocal phase shift of the dual-band CRLH metamaterial transmission lines with the use of schematic of dispersion diagrams.

**Figure 1:**
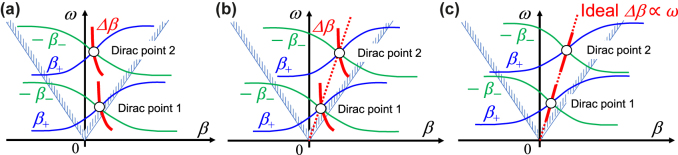
Schematic of typical dispersion diagrams of dual-band CRLH lines with two Dirac points and frequency dependence of the phase-shifting nonreciprocities Δ*β*. We here consider two conditions. Condition I is the case where both Dirac points in the lower and higher CRLH passbands are on the same linear line passing through the origin. Condition II is the case where both tangents of phase-shifting nonreciprocities Δ*β* at two Dirac points are proportional to the frequency. (a) General cases where neither condition I nor II is satisfied. (b) The case where only Condition I is satisfied. (c) The ideal case where both Conditions I and II are satisfied. For the special case, squint-free and unidirectional beam forming at dual-bands can be simultaneously realized in the leaky wave antennas by using ferrite-based nonreciprocal CRLH metamaterials.

In Figure 1, several examples of typical dispersion diagrams for dual-band nonreciprocal CRLH metamaterial lines with two Dirac points are shown. In general, sign of the slope of the dispersion curves is related to the group velocity and the transmitted power direction. Variables $\beta_{+}$ and $\beta_{-}$ in Figure 1 represent phase constants for dominant modes along the periodic CRLH line in the positive and negative propagation directions, respectively. Displacement of dispersion curves due to the nonreciprocity along the horizontal phase constant axis, defined here by a half the difference of the phase constants, is given by,

(1) $$\Delta \beta =\frac{\beta_{+}-\beta_{-}}{2}=\frac{\beta_{+}+\left(-\beta_{-}\right)}{2}$$

**Figure 2:**
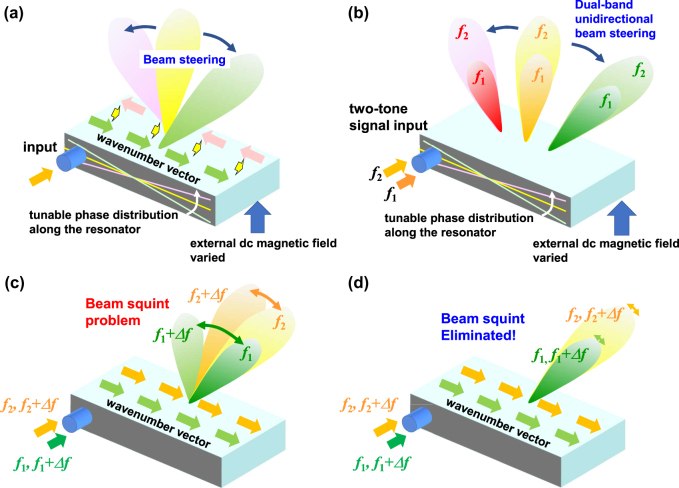
Schematic of beam scanning leaky wave antennas based on unidirectional traveling wave resonance using nonreciprocal CRLH metamaterial 1line. (a) Single-band beam scanning with a dynamic motion of the Dirac point in the dispersion diagram by changing the externally applied dc magnetic field. (b) Dual-band unidirectional beam scanning. (c) For the case of Figure 1b, beam directions at lower and higher bands can be set to the same direction but vary with the operating frequencies due to dispersive nonreciprocity Δ*β*. (d) The ideal case with dispersion-less nonreciprocity, as shown in Figure 1c, leads to dual-band unidirectional beam scanning as well as beam squint reduction.

In what follows, we call the quantity Δβ “phase-shifting nonreciprocity”. For reciprocal cases, $\beta_{+}$ = $\beta_{-}$ and Δβ = 0. In Figure 2, some examples of performances in dual-band unidirectional traveling-wave resonant leaky wave antennas using nonreciprocal CRLH metamaterial lines are shown. We consider two conditions for the nonreciprocity ∆*β*; I and II. Condition I is the case where both two Dirac points in the lower and higher CRLH passbands are on the same linear line passing through the origin. Condition II is another case where both tangents of phase-shifting nonreciprocities Δ*β* at the two Dirac points are proportional to the frequency with equivalent phase and group velocities at each Dirac point. Figure 1a shows a general case where neither condition I nor II is satisfied. When we select one of the intersections of the dispersion curves as an operating frequency of the size-independent unidirectional traveling-wave resonances, the resonator can achieve unidirectional leaky wave beam scanning at the frequency by changing the nonreciprocity Δ*β*, such as by changing the externally applied dc magnetic field to the ferrite, as shown in Figure 2a. Figure 1b denotes the dispersion diagram satisfying only condition I. In this case, dual-band unidirectional beam scanning may be realized, but when the operating frequency is deviated from the Dirac points, we will suffer from the beam squint problem, as shown in Figure 2c, due to the dispersive characteristics of the nonreciprocity Δ*β*. Figure 1c shows the special case where both conditions I and II are satisfied. For the special case, simultaneous satisfaction of conditions I and II ensures unidirectional beam scanning as well as beam squint elimination at dual-bands, as shown in Figure 2b and d.

## 3 Designed CRLH lines and demonstration

### 3.1 Equivalent circuit model and conditions for formation of two Dirac points

**Figure 3:**
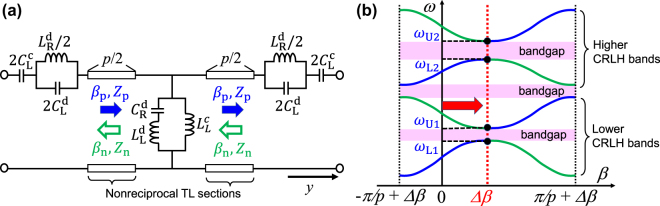
An equivalent circuit model and dispersion diagram for the nonreciprocal dual-band CRLH metamaterial lines. (a) The equivalent circuit model for the unit cell. (b) Typical dispersion curves without any Dirac points.

In this section, we briefly introduce how to construct two Dirac points in the dispersion diagram of nonreciprocal CRLH metamaterial lines and specifically how to design dispersion of the phase-shifting nonreciprocity proportional to the operating frequencies. Figure 3a shows an equivalent circuit model for the unit cell of the nonreciprocal CRLH metamaterial line along the *y* axis, and Figure 3b shows schematic of the dispersion curves without any Dirac points for general cases [27].

In Figure 3a, the length of the unit cell is *p*, and variables *C*_L_^c^, *C*_L_^d^, and *C*_R_^d^ denote capacitances of the designated capacitive elements, and *L*_R_^d^, *L*_L_^d^, and *L*_L_^c^ represent the inductances. The superscript “c” on the right-hand side of the variables stands for conventional inductive or capacitive elements used to construct single-band CRLH metamaterial lines, whereas the superscript “d” denotes elements additionally inserted in the unit cell to realize dual-band operation. In the unit cell, two identical nonreciprocal transmission line sections having wavevectors $\beta_{\text{p}}$ and $\beta_{\text{n}}$ and characteristic impedances $Z_{\text{p}}$ and $Z_{\text{n}}$ in the positive and negative *y* directions have also been introduced. In the present paper, the nonreciprocal line section is composed of a normally magnetized ferrite microstrip line, and the circuit parameters characterizing the sections are specifically determined by the gyro-magnetic characteristics of the ferrite and the geometrical configuration. The parameters including $\beta_{\text{p}}$ and $\beta_{\text{n}}$ must be previously given before finding solutions to the eigen modes for waves propagating along the periodic CRLH structures, based on Floquet’s theorem. The phase constants for the periodic structures correspond to the pair of variables $\beta_{+}$ and $\beta_{-}$ in Figure 1. Under the assumption that the characteristic impedances of the nonreciprocal transmission line sections show almost identical characteristics with $Z_{\text{p}}=Z_{\text{n}}=Z_{0}=1/Y_{0}$ , we have general dispersion curves with significantly large bandgaps, as shown in Figure 3b. To eliminate two bandgaps in the frequency regions from $\omega_{\text{L}1}$ to $\omega_{\text{U}1}$ and from $\omega_{\text{L}2}$ to $\omega_{\text{U}2}$ in Figure 3b and achieve the formation of two Dirac points at β=Δβ , as shown in Figure 1, the following two relations must be simultaneously satisfied in the same manner as reciprocal cases [9],

(2) $$\frac{Z_{\text{R}}^{\text{c}}}{Z_{\text{L}}^{\text{c}}}=\frac{Z_{\text{L}}^{\text{d}}}{Z_{\text{R}}^{\text{d}}},$$

(3) $$\omega_{\text{pse}}^{2}+\omega_{\text{C}0}^{2}=\omega_{\text{psh}}^{2}+\omega_{\text{L}0}^{2}$$

where

$$Z_{\text{R}}^{\text{c}}=Z_{0},\;Z_{\text{L}}^{\text{c}}=\sqrt{\frac{L_{\text{L}}^{\text{c}}}{C_{\text{L}}^{\text{c}}}},\;Z_{\text{L}}^{\text{d}}=\sqrt{\frac{L_{\text{L}}^{\text{d}}}{C_{\text{L}}^{\text{d}}}},\;Z_{\text{R}}^{\text{d}}=\sqrt{\frac{L_{\text{R}}^{\text{d}}}{C_{\text{R}}^{\text{d}}}},\;\;\omega_{\text{pse}}=\frac{1}{\sqrt{C_{\text{L}}^{\text{d}}L_{\text{R}}^{\text{d}}}}\text{, }\omega_{\text{psh}}=\frac{1}{\sqrt{C_{\text{R}}^{\text{d}}L_{\text{L}}^{\text{d}}}},$$

(4) $$\omega_{\text{C}0}=\sqrt{\frac{\bar{v}}{Z_{0}p}\left(\frac{1}{C_{\text{L}}^{\text{d}}}+\frac{1}{C_{\text{L}}^{\text{c}}}\right)},\;\omega_{\text{L}0}=\sqrt{\frac{\bar{v}}{Y_{0}p}\left(\frac{1}{L_{\text{L}}^{\text{d}}}+\frac{1}{L_{\text{L}}^{\text{c}}}\right)},\;\;\bar{v}=\frac{\omega }{\bar{\beta }},\;\bar{\beta }=\frac{\beta_{\text{p}}+\beta_{\text{n}}}{2},\text{ Δ}\beta =\frac{\beta_{\text{p}}-\beta_{\text{n}}}{2}$$

The frequency dependence of the nonreciprocity Δ*β* is expressed in terms of admittances of shunt stubs per unit cell, *Y*_1_ and *Y*_2_, that are asymmetrically inserted at left- and right-hand side strip edges, respectively, when looking at positive *y* direction, and is given by [23],

(5) $$\Delta \beta \left(\omega \right)\approx j\kappa \frac{\omega }{c}\frac{\left(Y_{1}-Y_{2}\right)}{2}\frac{h}{p}$$

with

(6) $$\kappa =\frac{\omega_{\text{m}}\omega }{\omega_{\text{h}}^{2}-\omega^{2}},\omega_{\text{h}}=\gamma \mu_{0}H_{0},\omega_{\text{m}}=\gamma \mu_{0}M_{S}$$

where it should be emphasized that the expression (5) explicitly shows the phase-shifting nonreciprocity originates from a combination of broken time-reversal and space-inversion symmetries, in that a factor κ in (5) is an off-diagonal component of the permeability tensor in ferrite and related to broken time-reversal symmetry, while another factor $\left(Y_{1}-Y_{2}\right)$ indicates the geometrical asymmetry of the waveguides due to broken space-inversion symmetry. In (5), *h* denotes the thickness of the substrate for the ferrite microstrip line, and in (6) the quantity $\omega_{\text{h}}$ represents the “magnetic resonance frequency” proportional to the internal dc magnetic field $H_{0}$ in the ferrite, γ is the gyromagnetic ratio, and $M_{S}$ the saturation magnetization.

In order to specify the frequency dependence of Δ*β* in (5), we assign the inductive element $L_{\text{L}}^{\text{c}}$ in the shunt branch in Figure 3a for *Y*_1_ and series LC resonant circuit including $L_{\text{L}}^{\text{d}}$ and $C_{\text{R}}^{\text{d}}$ for *Y*_2_. Then we have

(7) $$Y_{1}=\frac{1}{j\omega L_{\text{L}}^{\text{c}}},\;Y_{2}=\frac{1}{j\omega L_{\text{L}}^{\text{d}}+\frac{1}{j\omega C_{\text{R}}^{\text{d}}}}$$

For the weakly applied dc magnetic field to the ferrite where the operating frequency is selected at frequencies much higher than the magnetic resonance frequency with the relation $\omega \gg \omega_{\text{h}}$ , we have dispersive nonreciprocity Δ*β*, whose frequency dependence becomes complicated and looks like Figure 1a [29]. On the other hand, for the strongly applied dc magnetic field to the ferrite where the operational frequency is much lower than the magnetic resonance frequency with $\omega \ll \omega_{\text{h}}$ , the nonreciprocity Δ*β* (*ω*) in (5) reduces to

(8) $$\Delta \beta \left(\omega \right)\approx \frac{\mu_{0}}{2L_{\text{L}}^{\text{c}}}\frac{\omega_{\text{m}}}{\omega_{\text{h}}^{2}}\frac{h}{p}\omega$$

The above expression (8) clearly shows that the nonreciprocity is approximately proportional to the operational frequency *ω* both at lower and higher Dirac points that are located far below the magnetic resonance frequency $\omega_{\text{h}}$ , as ideally illustrated in Figure 1c.

### 3.2 Designed and fabricated circuit configuration

**Figure 4:**
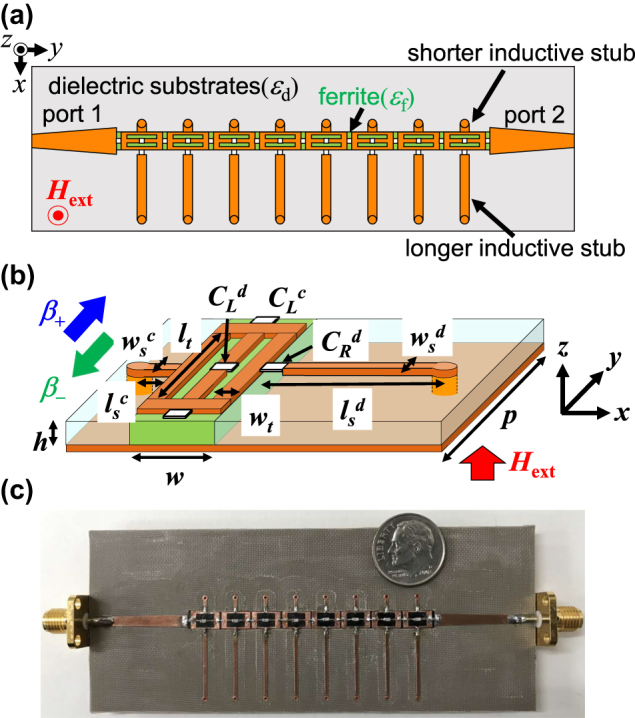
Schematic of the designed CRLH line and a photograph of the fabricated circuit for demonstration. (a) Top view of the designed CRLH line. (b) Perspective view of the unit cell. (c) Photo of the fabricated line.

In Figure 4, schematic of the designed CRLH metamaterial line, and photograph of the fabricated circuit are shown. As a commercial software, ANSYS HFSS ver. 19 was utilized in the numerical simulations for 3-D full-vector electromagnetic field analysis based on finite element method. Configuration parameters designed in the numerical simulations are given in the following: The heights of the ferrite and dielectric substrates are both *h* = 0.8 mm, their dielectric constants are *ε*_f_ = 15 and *ε*_d_ = 2.62. The length of the unit cell p = 6.9 mm, the width of the ferrite rod *w* = 4.0 mm, the length and width of the three parallel thin metallic strips in the center microstrip line are *l*_t_ = 4.5 mm and *w*_t_ = 0.3 mm. The length and width of the shorter inductive stub for *Y*_1_ on the left-hand side of Figure 4b, *l*_s_^c^ = 3.0 mm, *w*_s_^c^ = 1.0 mm. The length and width of longer inductive stubs made of the series LC tank for *Y*_2_ on the right-hand side, *l*_s_^d^ = 16.0 mm, and *w*_s_^d^ = 1.0 mm. The lumped-element capacitances *C*_L_^c^ = 2.1 pF, *C*_L_^d^ = 1.1 pF, and *C*_R_^d^ = 0.5 pF. We assume the saturation magnetization of the ferrite *μ*_0_ *M*_S_ = 175 mT, and the magnetic loss *μ*_0_Δ*H* = 5 mT. Since the Bloch impedance of the designed CRLH line was much smaller than 50 Ω, tapered feeding microstrip lines with the linearly varying widths were inserted between the designed CRLH line and 50-Ω input/output ports for impedance matching. Based on the designed configuration, we fabricated a prototype CRLH metamaterial line, as shown in Figure 4c. We utilized polycrystalline yttrium iron garnet (YIG) for the ferrite substrate, and a PTFE substrate (NPC-F260, Nippon Pillar Packing Co., Ltd.) was used for the dielectric substrate. In the measurement setup, the dc magnetic field was externally applied to the test circuits by using a neodymium magnet with the dimension of the length of 100 mm, width of 9.0 mm, and height of 50 mm that was placed just below a cupper plate for a ground plane, and the magnet was mechanically moved to adjust the desired magnitude of the external dc field.

### 3.3 Numerical simulation and experimental demonstration

**Figure 5:**
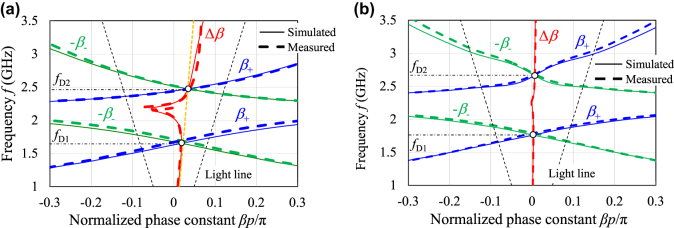
Dispersion diagrams and the phase-shifting nonreciprocities that are extracted from the transmission coefficients for the designed and fabricated dual-band CRLH metamaterial lines. (a) Nonreciprocal cases with the internal dc field *μ*_0_ *H*_0_ = 140 mT in the numerical simulation, and the externally applied dc magnetic field *μ*_0_ *H*_ext_ = 300 mT in the measurement. (b) Almost reciprocal cases with *μ*_0_ *H*_0_ = 400 mT in the numerical simulation and *μ*_0_ *H*_ext_ = 520 mT in the measurement.

Dispersion diagrams extracted from the simulated and measured phases of the transmission coefficients *S*_21_ and *S*_12_ as well as the phase shifting nonreciprocity Δ*β* are illustrated in Figure 5. Figure 5a corresponds to nonreciprocal cases with the phase shifting nonreciprocity Δβ≠0 , whereas Figure 5b corresponds to almost reciprocal cases with Δβ≈0 . In the numerical simulation set-up, the magnetic resonance frequency was selected at *f*_h_ = $\omega_{\text{h}}$ /2π = 3.92 GHz under the internal dc magnetic field of *μ*_0_ *H*_0_ = 140 mT for the nonreciprocal case in Figure 5a, while the magnetic resonance frequency was *f*_h_ = 11.2 GHz under the dc field of *μ*_0_ *H*_0_ = 400 mT for the almost reciprocal case in Figure 5b. In the measurement setup, the external dc magnetic field of *μ*_0_ *H*_ext_ = 300 mT was applied to the ferrite for Figure 5a, and *μ*_0_ *H*_ext_ = 520 mT for Figure 5b. Figure 5a and b clearly shows that dual passbands of the CRLH line are well designed in the numerical simulation and successfully reproduced in the measurement. It is found from Figure 5a that there are no significant bandgaps in the two CRLH passbands, and two Dirac cones are constructed with the intersection of the dispersion curves at *f*_D1_ = 1.64 GHz, Δ*βp*/*π* = 0.0188 in the lower passband and at *f*_D2_ = 2.47 GHz, Δ*βp*/*π* = 0.0339 in the higher passband. In addition, these intersections of the curves are found to be located in the vicinity of the same linear line with the slope of the refractive index of 0.27. It is noted that there exists a singularity in the nonreciprocity Δ*β* around at 2.2 GHz within a band gap between lower and higher CRLH passbands, as seen in Figure 5a. The singularity is likely to disturb the formation of linearity over the wideband region in the phase-shifting nonreciprocity. Figure 5b shows a considerably small nonreciprocities Δ*β* both in the numerical simulation and measurement due to the magnetic resonance frequency *f*_h_ much higher than the operational frequency.

**Figure 6:**
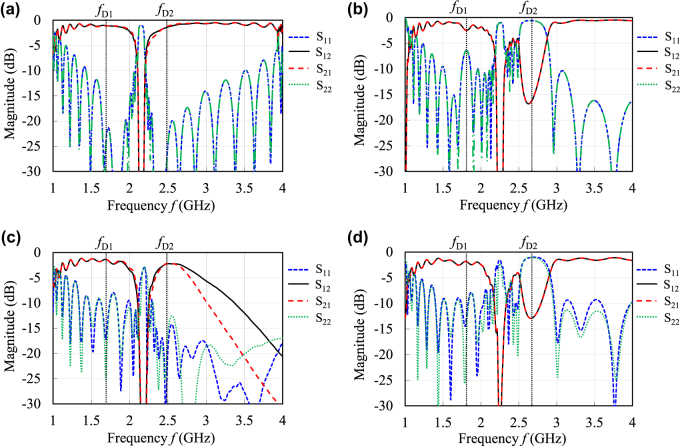
Magnitudes of transmission and reflection coefficients of the designed and fabricated CRLH lines. (a) and (b) denote numerical simulation results for nonreciprocal and almost reciprocal cases, respectively. (c) and (d) represent the measurement results corresponding to (a) and (b), respectively. (a) The internal dc field *μ*_0_ *H*_0_ = 140 mT. (b) *μ*_0_ *H*_0_ = 400 mT. (c) The external dc magnetic field *μ*_0_ *H*_ext_ = 300 mT. (d) *μ*_0_ *H*_ext_ = 520 mT.

In Figure 6, magnitudes of the simulated and measured transmission and reflection coefficients are plotted. Figure 6a and b shows the numerical simulation results and Figure 6c and d does the measurement results. Figure 6a and c denotes the nonreciprocal cases corresponding to Figure 5a, and Figure 6b and d illustrates almost reciprocal cases corresponding to Figure 5b. It should be emphasized that simulated magnitudes of the transmission coefficients *S*_21_ and *S*_12_ in Figure 6a and b show almost identical characteristics, regardless of whether the designed CRLH lines are nonreciprocal or not. In Figure 6c, the transmission coefficients *S*_21_ and *S*_12_ at higher frequencies were found to be seriously attenuated because the dc magnetic field applied was nonuniform in the ferrite. We have confirmed that by replacing the permanent magnet with an electromagnet in order to apply a uniform dc field to the ferrite, both the measured transmission coefficients *S*_21_ and *S*_12_ successfully recovered with greater than −3 dB and almost identical transmission in the frequency region from 2.4 to 3.6 GHz. In addition, a big notch is found around at 2.7 GHz in Figure 6b and d for almost reciprocal cases with sufficiently large dc field applied to the ferrite. This is because the Dirac point in the higher CRLH passband collapsed, and a bandgap appears due to the impedance-mismatching in which effective permeability of the CRLH line has been considerably changed by enhancing the applied dc magnetic field.

**Figure 7:**
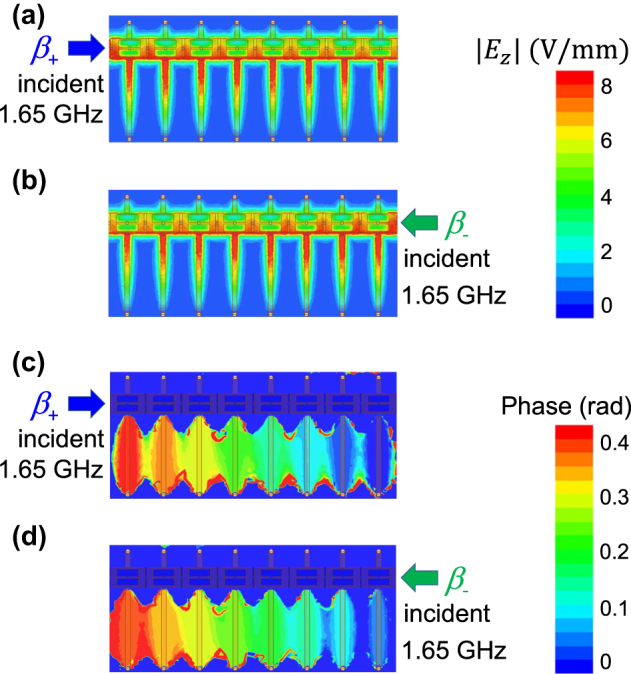
Magnitude and phase distribution of the electric field component *E*_z_ observed on a plane a half the substrate thickness above the ground plane at 1.65 GHz. (a), (b) Magnitude. (c), (d) Phase distribution. Signal incidence (a), (c) from the left port 1 and (b), (d) from the right port 2.

**Figure 8:**
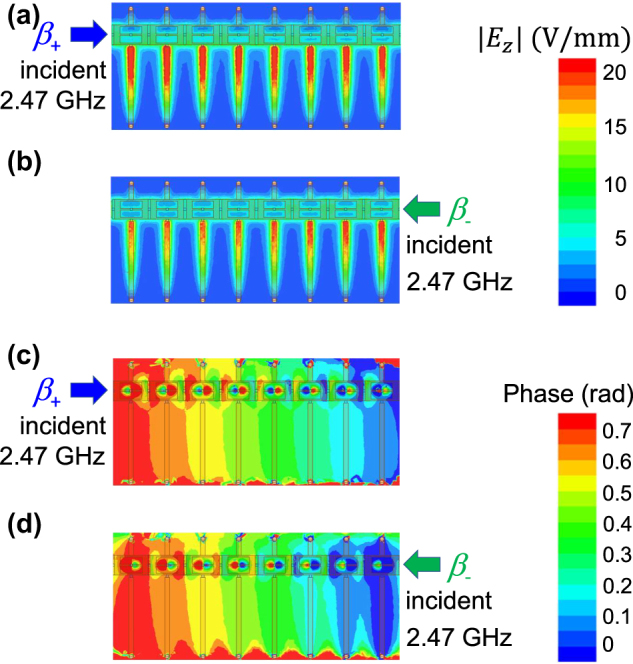
Magnitude and phase distribution of the electric field component *E*_z_ observed on a plane a half the substrate thickness above the ground plane at 2.47 GHz. (a), (b) Magnitude. (c), (d) Phase distribution. (a), (c) Signal incidence from left port 1 and (b), (d) from right port 2.

To confirm the validity of the simulated dispersion curves in Figure 5a, simulated magnitude and phase profiles of the electric field component *E*_z_ along the transmission line are observed around at the Dirac points in the lower and higher CRLH passbands and plotted in Figures 7 and 8, respectively. Figures 7 and 8 represent the field profiles measured at 1.65 and 2.47 GHz. The phase distribution in Figure 7c clearly shows the dominant forward wave mode propagation with wavenumber vector parallel to the transmitted power direction, whereas that in Figure 7d represents the backward wave mode propagation with the wavenumber vector antiparallel to the transmitted power direction. It is found from Figure 7c and d that their phase gradients in the longitudinal *y* direction are found almost the same. Figure 8 is also the case similar to Figure 7. Therefore, the observed field profiles in Figures 7 and 8 clearly show the wave propagation around at Dirac points with nonzero phase constants. We can also confirm the validity of the simulation results from the rough estimate of phase gradients along the line in Figures 7 and 8. Figure 7c and d shows that the phase difference over the 8-cell CRLH line was 0.4 rad and then Δ*β*p = 0.05 rad/cell, which corresponds to beam angle of *θ* = sin^−1^(Δ*β*/*β*_0_) = 12.5° that was measured with respect to the normal to the planar line, where *β*_0_ is the phase constant in vacuum. On the other hand, Figure 8c and d denotes the phase difference over the line was 0.7 rad and then Δ*β*p = 0.0875 rad/cell, corresponding to the beam angle of 14.6°. Therefore, we have confirmed that the beam directions estimated from the transmission characteristics of the designed nonreciprocal CRLH metamaterial lines at two different frequencies are found to be almost the same. Thus, we have successfully demonstrated the transmission of dual band nonreciprocal CRLH metamaterial lines having two Dirac points with phase-shifting nonreciprocity approximately proportional to the operating frequency.

## 4 Conclusions

In this work, we have proposed and demonstrated dual-band nonreciprocal CRLH metamaterial lines with the phase-shifting nonreciprocity approximately proportional to the operating frequency by using normally magnetized ferrite microstrip lines. The nonreciprocity was dynamically controlled by changing the externally applied dc magnetic field. Dynamic change in the nonreciprocal phase gradient along the line will enable us to realize dual-band, unidirectional, and squint-free beam-scanning leaky-wave antennas. A prototype nonreciprocal metamaterial line using polycrystalline yttrium iron garnet was fabricated and the fundamental transmission characteristics were measured for verification of our basic concept. In the near future, the proposed nonreciprocal CRLH metamaterial lines will be developed further and implemented in multi-band, unidirectional, squint-free beam-scanning leaky wave antennas as well as other new applications. Of course, the basic concept and design procedure treated in the paper are scalable and not limited in the microwave/millimeter-wave technologies. They will also be developed in the terahertz as well as optical regions for potential applications to multifunctional beam-steering antennas based on nanotechnologies.
